# Construction of Symmetric Flexible Electrochromic and Rechargeable Supercapacitors Based on a 1,3,6,8-Pyrenetetrasulfonic Acid Tetrasodium Salt-Loaded Polyaniline Nanostructured Film

**DOI:** 10.3390/ma18122836

**Published:** 2025-06-16

**Authors:** Yi Wang, Ze Wang, Zilong Zhang, Yujie Yan, An Xie, Tong Feng, Chunyang Jia

**Affiliations:** 1 Key Laboratory of Functional Materials and Applications of Fujian Province, School of Materials Science and Engineering, Xiamen University of Technology, Xiamen 361024, China; yiwang@xmut.edu.cn (Y.W.); 2322161045@stu.xmut.edu.cn (Z.W.); 2222031544@stu.xmut.edu.cn (Z.Z.); yujieyan@xmut.edu.cn (Y.Y.); anxie@xmut.edu.cn (A.X.); 2 National Key Laboratory of Electronic Thin Films and Integrated Devices, National Engineering Research Center of Electromagnetic Radlation Control Materials, School of Integrated Circuit Science and Engineering, University of Electronic Science and Technology of China, Chengdu 610054, China; 3 School of Mechanical Electrical and Information Engineering, Xiamen Institute of Technology, Xiamen 361021, China

**Keywords:** multi-functions, electrochromic, PANI, doping, supercapacitor

## Abstract

Electrochromic supercapacitors (ECSCs), which visually indicate their operating status through color changes, have attracted considerable attention in the field of wearable electronics. The conductive polymer polyaniline (PANI) shows great potential for integrated intelligent devices by combining bi-functional electrochromic spectral modulation and energy storage capabilities. In this work, a microsphere-like structured PANI-based composite film was fabricated on a porous Au/nylon 66 electrode via a one-step electrochemical copolymerization process, using 1,3,6,8-pyrenetetrasulfonic acid tetrasodium salt (PTSA) as both the dopant and cross-linking agent for the PANI backbone, serving as the ECSC electrode. Compared to the pristine PANI electrode, the PANI-PTSA composite film exhibits lower intrinsic resistance and higher electrical conductivity, delivering a higher specific capacitance of 310.0 F g⁻^1^@1 A g⁻^1^ and an areal capacitance of 340.0 mF cm⁻^2^@1 mA cm⁻^2^, respectively. The dopant facilitates enhanced electrochemical performance by promoting charge transport within the PANI polymer network. Meanwhile, as a counter anion to the PANI backbone, PTSA regulates the growth of PANI chains and acts as a morphological controller. Furthermore, a symmetric ECSC based on the PANI-PTSA_8:1_ electrode was assembled, and its electrochemical properties were thoroughly investigated. The device demonstrated a high specific capacitance of 169.2 mF cm⁻^2^ at 1 mA cm⁻^2^, a notable energy density of 23.5 μWh cm⁻^2^ at a power density of 0.5 mW cm⁻^2^, and excellent cycling stability with 79% capacitance retention after 3000 cycles at a current density of 5 mA cm⁻^2^, alongside remarkable mechanical flexibility. Additionally, the working status of the ECSCs can be directly monitored through reversible color changes from yellow-green to deep blue during charge–discharge processes.

## 1. Introduction

Electrochromism refers to the unique property of certain materials to reversibly change their optical characteristics—such as transmittance, absorbance, or reflectance—under an applied voltage. These materials have attracted considerable attention in recent decades due to their broad range of potential applications, including electronic displays, smart windows, anti-glare rearview mirrors, and more [1,2,3]. The rapid development of portable, wearable, and implantable electronics, as well as the increasing demand for novel functionality, has stimulated the development of flexible electrochromic devices (ECDs), with more emphasis on novel features such as intelligent, multi-functional, and integrated functions [4,5]. This calls for innovative technological solutions for developing “smart” ECDs.

Developing new functions in the process of fabricating remarkable ECDs is crucial to the intellectualization of the devices [6,7,8]. The typical ECD structure is a multi-layer sandwich structure comprised of an electrochromic (EC) layer, an ion storage layer, an electrolyte layer, and two conducting electrodes [9]. In particular, the ECD also can be regarded as an electrochemical thin-film energy storage device, which is generally associated with electrochemical reactions of the electrode materials and possess sandwich device structures [5,10]. Therefore, integrating electrochromic and energy storage functions into a single device is highly desirable for multi-functional applications. With its ability to change color depending on the energy storage state, this multipurpose gadget offers several benefits. It may give an early warning before shutting down based on visible color variation, increasing convenience and working efficiency [11,12].

One of the main challenges in developing multi-functional electrochromic–energy storage devices lies in identifying suitable materials and fabrication methods that enhance simplicity and cost-effectiveness. In recent years, conducting polymers have been extensively studied for use in both electrochromic devices and supercapacitors [13]. Polyaniline (PANI) is the most studied conducting polymer as an electrode material in supercapacitors and a chromic material in ECDs due to its intrinsic pseudocapacitive properties, high conductivity, cost-effectiveness, and reversible color-changing characteristics (between light yellow, green, and blue) [14,15]. Chen et al. [16] synthesized carbon quantum dot-doped PANI via spray-coating, achieving enhanced capacitive and electrochromic performance. Wu et al. [17] employed galvanostatic electropolymerization to prepare PANI films with superior electrochromic and energy storage properties. Xu et al. [18] fabricated a 3D hierarchical PANI/tungsten trioxide (WO_3_) composite through in situ intercalation, exhibiting improved electrochromic stability due to optimized interlayer spacing and increased ion storage capacity. Typically, the intrinsic characteristics of PANI are determined by its oxidation state, dopant type, and extent of doping. To improve the electrical conductivity of PANI, inorganic acids like HCl, H_2_SO_4_, HNO_3_, and HClO_4_ are typically utilized as dopants [19]. These strong acids are extremely corrosive by nature, but they can also produce secondary growth or aggregation of polymer chains and coiling of the chains due to kinetic and thermodynamic barriers [20,21]. Furthermore, the coiling of polymer chains generates localized polarons, which significantly hinders inter-chain and intra-chain hopping processes—an issue particularly critical for electrochemical reactions. To address this challenge and further enhance the electrochemical performance of PANI, the introduction of organic molecules as co-doping agents has proven to be an effective strategy. To drive redox reactions and balance the charge produced during the coloring, bleaching, or charging/discharging processes, the dopant counterions can intercalate or deintercalate the polymer chain [20]. 1,3,6,8-pyrenetetrasulfonic acid tetrasodium salt (PTSA), a kind of organic salt containing -SO_3_^−^ groups, is readily available, inexpensive, and water-soluble [22]. The PTSA, acting as ionic dopant and cross-linking agent, can be assembled onto the charged PANI backbone via the electroatatic interactions. This interaction between organic salt and PANI can also play a critical role in deciding the conductivity, chain growth, and morphology regulator [23]. Furthermore, PTSA has a large conjugated structure, which can increase the rigidity of the molecule, making the prepared PANI polymer network more stable [24]. Hence, due to the excellent cross-linking and ionic doping effect of PTSA, it is expected to result in a high-performance and multi-functional device. However, only a few studies have focused on the effect of larger conjugated structural dopants on PANI-based multi-functional devices.

In this study, we reported an effective approach for using PTSA as an ionic dopant and cross-linking agent to improve the electrochemical performance of the PANI film. The PANI film electrodes were created using a one-step co-deposition technique, which has the benefit of being simple to fabricate and very efficient. The PANI chains are cross-linked, enabled by the -SO_3_^−^ groups of PTSA molecular by electroatatic interactions for the PANI backbone, which will affect the morphology and electrochemical properties of film. The inclusion of PTSA as a dopant during the process resulted in nanostructured materials with superior electrochemical performance and stability. The PANI-PTSA_8:1_ film electrode demonstrates a better electrochemical energy storage characteristic compared to the pristine PANI film (340.0 mF cm^−2^ at 1 mA cm^−2^ and specific capacitance of 310.0 F g^−1^ at 1 A g^−1^ for the PANI-PTSA_8:1_ electrode). The as-fabricated symmetrical device demonstrates a superior multi-function of energy storage and electrochromic spectral modulation. The device achieves a high area capacitance of 169.2 mF cm^−2^ at 1 mA cm^−2^ and a considerable energy density of 23.5 μWh cm^−2^ at 0.5 mW cm^−2^. The fabricated multi-functional device exhibits excellent flexibility and long cycling stability. Notably, its reversible color change provides a promising visual indicator of the energy storage level, enabling real-time monitoring with the naked eye.

## 2. Experimental Section

### 2.1. Materials

Aniline (ANI, AR, ≥99.5%), 1,3,6,8-pyrenetetrasulfonic acid tetrasodium salt (PTSA, ≥97%), polyvinyl alcohol (PVA, hydrolyzed, and medium molecular weight), and perchloric acid (HClO_4_, 70.0–72.0%) were obtained from Shanghai Aladdin Biochemical Technology Co., Ltd. (Shanghai, China). Sulfuric acid (H_2_SO_4_, 98 wt%) was supplied by Tianjin ZhiYuan Reagent Co., Ltd. (Tianjin, China). Prior to use, the aniline monomer was purified via vacuum distillation. Deionized water (DIW) was used in all experimental procedures.

### 2.2. Preparation of the Au/Nylon 66 Porous Film

A thin layer of gold was thermally evaporated onto the nylon 66 membrane with a pore diameter of 0.45 μm. During the deposition process, the vacuum chamber was first evacuated to a base pressure of 3 × 10⁻^3^ Pa. A photograph of the large Au/nylon 66 film electrode is presented in Figure S3.

### 2.3. Electrodeposition of the Polymer Film

The PANI-PTSA film was deposited onto the Au/nylon 66 porous substrate from an aqueous solution containing the aniline monomer and PTSA as the electrolyte. The electrolyte was prepared by dissolving 0.1 M HClO_4_, ANI (0.1 M), and PTSA in the aqueous solution. The following molar ratios of ANI:PTSA were used: 10:1, 8:1, 5:1, and 3:1. The initial electrodeposition step consisted of cycling the potential five times between 0 and 1.0 V versus Ag/AgCl. After rinsing with deionized water (DIW), electrodeposition was performed at a current density of 0.25 mA cm⁻^2^ for 8000 s. The resulting film was then washed with DIW and dried under vacuum at 60 °C for 12 h. For comparison, a pristine PANI film was also prepared as a reference using the same procedure but without PTSA.

### 2.4. Fabrication of Flexible Supercapacitors

A flexible supercapacitor was fabricated by sandwiching two symmetric PANI-based Au/nylon 66 film electrodes with a PVA/H_2_SO_4_ gel electrolyte. The gel electrolyte was prepared by vigorously stirring a mixture of PVA (3 g), H_2_SO_4_ (3 g), and deionized water (30 mL) at 85 °C until a clear viscous solution was formed [25]. The device was left at room temperature under ambient conditions to allow excess water to evaporate. Finally, a polyethylene (PE) film was applied as an encapsulation layer over the entire device.

### 2.5. Characterization

Morphologies of the materials were characterized using a scanning electron microscope (SEM, GeminiSEM 300, Carl Zeiss, Oberkochen, Germany). X-ray photoelectron spectroscopy (XPS) measurements were conducted using a Kratos XSAM800 instrument (Kratos Analytical Ltd., Manchester, UK). The electrochemical performance of a single electrode, serving as the working electrode, was evaluated in a three-electrode setup with a CHI 660E electrochemical workstation (CH Instruments, Shanghai, China) in 1 M H_2_SO_4_ aqueous solution. Platinum foil and Ag/AgCl electrodes were employed as the counter and reference electrodes, respectively. Electrochemical tests of the encapsulated device were performed in a two-electrode configuration. Electrochemical impedance spectroscopy (EIS) measurements were carried out over a frequency range of 10⁻^2^ to 10^5^ Hz. Galvanostatic charge–discharge (GCD) curves were recorded using the electrochemical workstation. The spectral reflectance of the device was measured with a PerkinElmer Frontier FT-IR spectrometer (PerkinElmer, Waltham, MA, USA).

### 2.6. Calculation Methods of the Electrochemical Date

The gravimetric specific capacitance (*Cs*) and areal capacitance (*Ca*) were determined from the GCD profiles according to the following formulas, respectively [26,27,28]:

$$C_{S}=\frac{I\times ∆t}{m\times ∆V}\;\;(F\;g^{-1})$$

$$C_{a}=\frac{I\times ∆t}{a\times ∆V}\;\;(\mathrm{mF}{\mathrm{cm}}^{-2})$$

where I is the discharge current (mA), ∆t is the discharge time (s), ∆V is the potential window (V), m is the mass of the active electrode materials (mg), and a represents the geometry of the electrode or device (cm^2^). Moreover, the energy density (E, μWh cm^−2^) and the power density (P, μWh cm^−2^) of as prepared supercapacitors can be expressed as [14]:

$$E=\frac{1}{2\times 3.6}C∆V^{2}\;\;(\mu \mathrm{Wh}{\mathrm{cm}}^{-2})$$

$$P=\frac{3.6E}{∆t}(\mathrm{mW}{\mathrm{cm}}^{-2})$$

## 3. Results and Discussion

**Figure 1:**
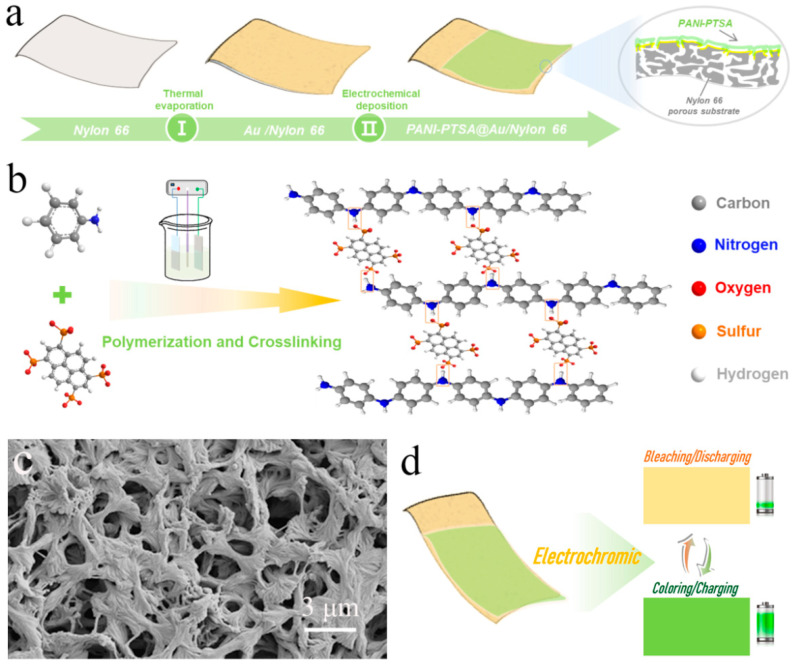
Schematic representation and characterization of the flexible PANI-PTSA@Au/nylon 66 film electrode with electrochromic and energy storage functionality. (**a**) Schematic illustration of the preparation process of the PANI-based film electrode. (**b**) Schematic illustration of the doping and cross-linking interaction between PANI and PTSA. (**c**) SEM image of the Au/nylon 66 porous substrate. (**d**) A demonstration of the flexible PANI-PTSA film with electrochromism and energy storage function.

We adopted Nylon 66 porous substrate as the conductive substrate (denoted as Au/nylon 66, as shown in Figure 1a), depositing metallic gold nanoparticles by thermal evaporation technology. PTSA-doped polyaniline (denoted as PANI-PTSAx:y; the x, y refers to the mole ratio of aniline monomer to PTSA) was prepared via the electropolymerization of aniline in HClO_4_ electrolytes containing a certain amount of PTSA. In the Discussion section, PANI@Au/nylon 66 and PANI-PTSAx:y@Au/nylon 66 are denoted as PANI and PANI-PTSAx:y, respectively. Figure 1b illustrates the schematic of doping PTSA for PANI. Since the PTSA molecule has numerous -SO_3_^−^ groups that can react with various PANI chains to build network structures, it was used as both an ionic dopant and a cross-linker via tethering nitrogen groups on PANI polymer chains [23]. Figure 1c shows the morphology of the Au/nylon 66 porous structure, which consists of a network of interwoven fibers and numerous micron-sized voids, resulting in a high specific surface area. These voids can serve as “ion-buffering reservoirs,” effectively shortening the ion diffusion pathways [29,30]. The PANI film was simultaneously applied to the current collector’s surface (Figure 1d). The film was also in the outer layer of the dual-functional device when the device was assembled. This method significantly enhances the observation of the color shift of the multi-functional gadget in this study [31,32].

**Figure 2:**
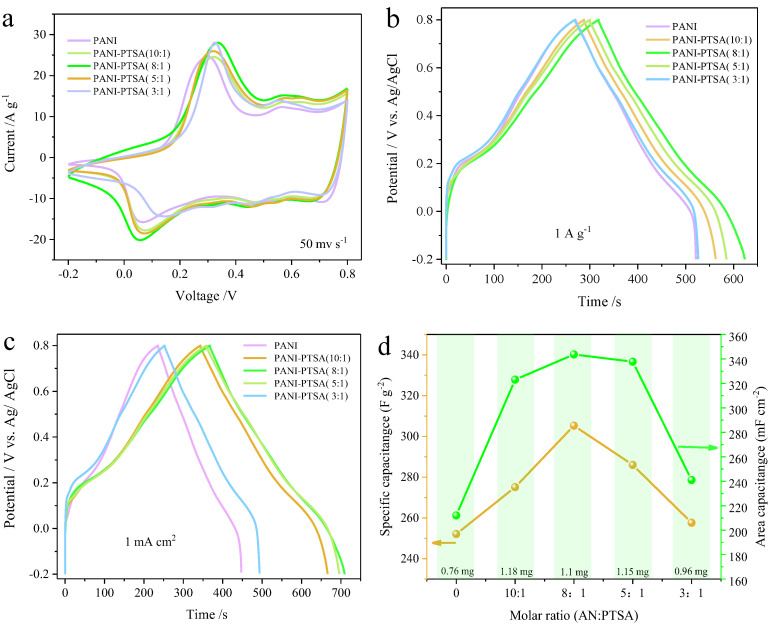
Electrochemical performance of composite electrodes with different PTSA doping weights. (**a**) CV curves, (**b**,**c**) GCD evolution curves with various dopant weights, and (**d**) area and specific capacitance summary plots of GCD.

Figure 2a illustrates typical cyclic voltammetry (CV) curves, including PANI and PANI-PTSAx:y with different doping ratios in 1 M H_2_SO_4_ solutions. The strong redox peaks were found in all electrodes due to the reversible faradaic reactions of PANI, followed by doping/dedoping [33,34]. Comparatively, the enclosed area of CV curves for the PANI-PTSA_8:1_ film electrode is greater than that of the original PANI and other doped ratio samples, showing a higher pseudocapacitance of the electrode. Figure 2b displays the galvanostatic charge–discharge (GCD) curves of the PANI electrode with various PTSA doping ratios in the composite electrodes. With an increase in the PTSA doping ratio, the discharge time and specific capacitance increase gradually, and the PANI-PTSA_8:1_ has the largest capacitance of 310 F g^−1^ at 1 A g^−1^. With a further increase in PTSA content, the capacitance of the PANI-PTSA sample is lower than that of PANI-PTSA_8:1_. Therefore, the PANI-PTSA_8:1_ sample exhibits the best electrochemical performance. The GCD curves in Figure 2c were also used to compute the areal capacitances of the PANI-based electrode. The PANI-PTSA_8:1_ sample had the best areal capacitance value, 340.0 mF cm^−2^, at 1 mA cm^−2^. The corresponding pseudocapacitance values of PANI and the PANI-PTSAx:y composite are shown in Figure 2d.

**Figure 3:**
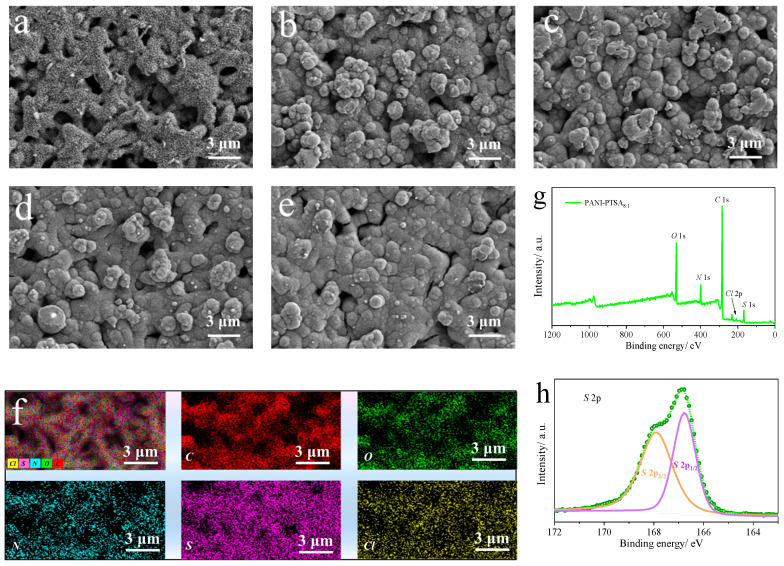
Morphological and compositional characterization of PANI and PANI-PTSA composites with varying PTSA ratios. SEM images illustrate the surface morphology of (**a**) pristine PANI, (**b**) PANI-PTSA_10:1_, (**c**) PANI-PTSA_8:1_, (**d**) PANI-PTSA_5:1_, and (**e**) PANI-PTSA_3:1_. (**f**) EDS elemental mapping of PANI-PTSA_8:1_ confirms the uniform distribution of key elements. (**g**) The XPS wide-scan spectrum of PANI-PTSA_8:1_ reveals the elemental composition, while (**h**) the deconvoluted S 2p spectra provide detailed insights into the sulfur chemical states within the composite.

Furthermore, SEM was used to examine the morphologies of PANI-based film samples generated at different doping rates. The PANI film produced in HClO_4_ solution without the addition of PTSA exhibits a linked nanofiber morphological structure, as seen in Figure 3a. Similar morphologies can also be found in the literature [35,36]. However, once the sulfonic-based organic ionic dopant was added, the PANI-PTSA film showed different morphology features. As can be observed from Figure 3b–e, the microspheres were arranged in a compacted manner on the surface of the Au/nylon 66 fibers. The morphology of the film is influenced by the presence of PTSA, whose sulfonate groups act as dispersing agents for aniline monomers in the deposition solution [24]. Furthermore, in the presence of PTSA during PANI synthesis, the polymer chains tend to form microsphere-like nanostructures, possibly due to enhanced π–π interactions with the aromatic counter anions of PTSA [37]. As the doping concentration of PTSA increases (Figure 3b–e), the deposited film becomes denser, and the microsphere size enlarges. The enhanced electrochemical and capacitive performance of the PANI-PTSA_8:1_ film electrode can thus be attributed to improved ion and electron transport, resulting from the synergistic effect of the optimized morphology and doping ratio. The energy-dispersive X-ray spectroscopy (EDS) elemental mapping of the PANI-PTSA_8:1_ film reveals the presence of carbon (C) and nitrogen (N) from the polymer backbone, as well as oxygen (O) and sulfur (S) from the PTSA counter ions. This indicates that the PTSA molecules and PANI form composite films via electrostatic interactions. In addition, the XPS spectra were obtained to evaluate the PANI-PTSA_8:1_ film surface element compositions and doping level. As shown in Figure 3g, the presence of a sulfide peak in PANI-PTSA_8:1_ implies that the PANI polymer backbone was doped with the -SO_3_^−^ originating from the PTSA molecule during polymerization. Chlorine is also observed in the full spectrum (the doping inorganic acid is perchloric acid), indicating that PANI was also doped with perchlorate. Figure 3h shows the XPS spectra of S 2p, originating from -SO_3_^−^ ions doped into the PANI polymer backbone, fitting with the typical spin-orbit doublets of -SO_3_^−^ groups, including S 2p3/2 and S 2p1/2 [38]. These results confirm the chemical composition for the PANI-PTSA_8:1_ film sample.

**Figure 4:**
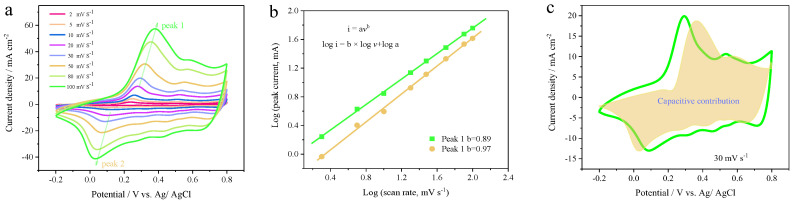

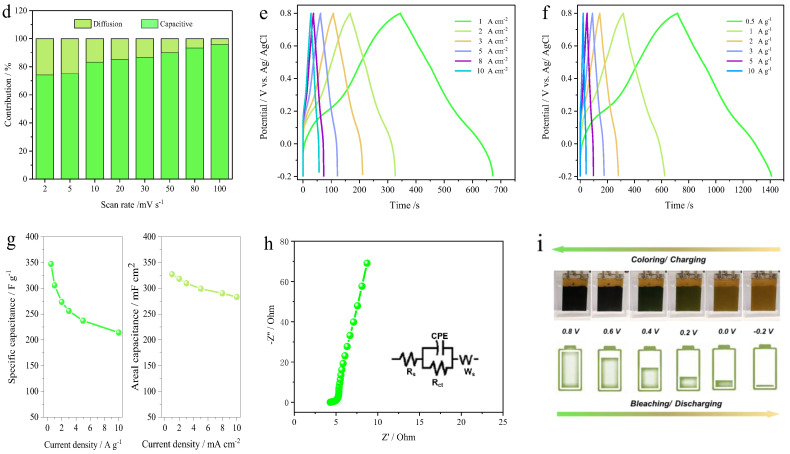
Electrochemical performance and electrochromic behavior of the PANI-PTSA8:1 film. (**a**) CV curves recorded at various scan rates. (**b**) The relationship of log (peak current) and log (scan rate) for the PANI-PTSA_8:1_ film. (**c**) Quantitative analysis of the capacitive contribution at 50 mV s⁻^1^ in 1 M H_2_SO_4_ electrolyte, with (**d**) capacitive contribution ratios evaluated across different scan rates. (**e**,**f**) GCD curves showing areal and specific capacitances at multiple current densities, respectively, with (**g**) summary plots comparing areal and specific capacitances derived from GCD measurements. (**h**) EIS profile highlighting the film’s charge transfer resistance and ion diffusion characteristics. (**i**) Digital photographs and schematic illustrations capture the color change of the film at various charge/discharge states.

Further CV measurements of the PANI-PTSA_8:1_ film were carried out, ranging from 2 to 100 mV s^−1^. The nonrectangular shape of the CV profiles, as seen in Figure 4a, may be related to the PANI electrode’s pseudocapacitive properties. The cyclic voltammetry (CV) curve exhibits two broad redox peaks, indicating the sequential redox transitions from leucoemeraldine to emeraldine salt and from emeraldine salt to pernigraniline, which are characteristic of the pseudocapacitance exhibited by the PANI electrode [39]. Moreover, the shape of the CV curve is well maintained with increasing scan rates, indicating that PANI doped with PTSA exhibits low internal resistance and rapid redox kinetics. To gain deeper insight into the charge storage mechanisms and electrochemical performance of the PANI-PTSA electrode, further kinetic analysis was performed based on the CV profiles. According to previous studies, the current density (*i*) and scan rate (*v*) of supercapacitor electrodes follow the empirical relationship *i* = *kv^b^*, where *k* and *b* are adjustable parameters [40,41]. The *b* value is an important parameter for the electrochemical kinetics, ranging from 0.5 to 1. When the *b* value approaches 0.5, the slow diffusion-limited process dominates the charge storage, whereas when the *b* value approaches 1, it implies that the process is surface-controlled (fast capacitive behavior) [6,42]. Based on the plot of log *i* versus log *v*, the *b* values were calculated to be 0.89 and 0.97, respectively, indicating that the energy storage process is predominantly governed by capacitive-controlled behavior (Figure 4b) [15]. Furthermore, Dunn’s method can be used to determine the relative contribution of the diffusion-controlled process and capacitance effect quantitatively. Figure 4c presents the capacitive contribution (of about 86.7%) to the total charge storage (light yellow area), measured based on the PANI-PTSA electrode at a scan rate of 30 mV s^−1^. The capacitive contribution of the PANI-PTSA electrode is dominant, indicating the fast surface kinetics process. Furthermore, Figure 4d shows the relative contributions of capacitive and diffusion-controlled processes at various scan rates, with capacitive contributions of 74.2%, 75.1%, 83.3%, 85.2%, 86.7%, 90.0%, 93.3%, and 95.8% corresponding to scan rates of 2, 5, 10, 20, 30, 50, 80, and 100 mV s^−1^, respectively. This increasing trend indicates that the surface-controlled capacitive behavior becomes more dominant at higher charge–discharge rates. It also reflects the fast ion diffusion kinetics in the PANI-PTSA8:1 film, which contributes to its excellent performance. GCD curves of the PANI-PTSA_8:1_ electrode were recorded at different current densities (Figure 4e,f). GCD profiles exhibit the typical triangle-shape pattern with a negligible IR drop, indicating highly reversible electrochemical storage behavior and good conductivity of the PANI film and Au@Nylon electrode. The areal capacitance and specific capacitance of the PANI-PTSA_8:1_ film electrode calculated from GCD curves is summarized in Figure 4g. This film exhibits a notable specific capacitance of 350.0 F g⁻^1^ at a current density of 0.5 A g⁻^1^ and 214.0 F g⁻^1^ at 10 A g⁻^1^, demonstrating moderate rate capability with 61% capacitance retention as the current density increases twentyfold. The areal capacitances of the PANI-PTSA_8:1_ electrode, derived from GCD curves, are 333.0 mF cm⁻^2^ at 1 mA cm⁻^2^ and 283.0 mF cm⁻^2^ at 10 mA cm⁻^2^, respectively. In comparison, the pure PANI film shows a specific capacitance of 260.8 F g⁻^1^ at 0.5 A g⁻^1^ and an areal capacitance of 211.3 mF cm⁻^2^ at 1 mA cm⁻^2^ (Figure S1). These results demonstrate that incorporating dopants such as PTSA into the polymer chain effectively enhances the pseudocapacitive performance of PANI.

Additionally, the electrochemical kinetics and ionic resistance were investigated using the electrochemical impedance spectroscopy (EIS) measurement [25]. In Figure 4h, the standard Nyquist plots for the PANI-PTSA_8:1_ electrodes are shown. From the intersection of the real axes in the Nyquist plots, the equivalent series resistance (Rs) for the PANI-PTSA_8:1_ electrodes is 3.4 Ω, suggesting that the PTSA/PANI composites have relatively low internal resistance. It is possible to determine the charge transfer resistances (Rct) of the PANI-PTSA_8:1_ electrodes from the visible small semicircle in the high frequency zone, which is 1.8 Ω. The PANI network produced with PTSA as a dopant has strong conductivity and rapid electron transport on interfaces because of the redox reactions, according to the EIS result [26]. Figure 4i clearly illustrates the color change of the PANI-PTSA film in response to varying applied voltages, indicating that the color evolution can be utilized for the real-time monitoring of the film’s operating states. At a potential of –0.2 V, the film appears light yellow (influenced by the Au/nylon 66 substrate). When the charging voltage increases to 0.5 V, the color shifts from light yellow to light green. Then, the film changes to green as the charging continues and reaches dark green in the nearly fully charged state, derived from the decreased transmittance of the PANI film. These results demonstrate that the supercapacitor’s energy storage level can be approximately monitored through its color changes, offering a novel user experience for intelligent wearable electronic devices.

**Figure 5:**
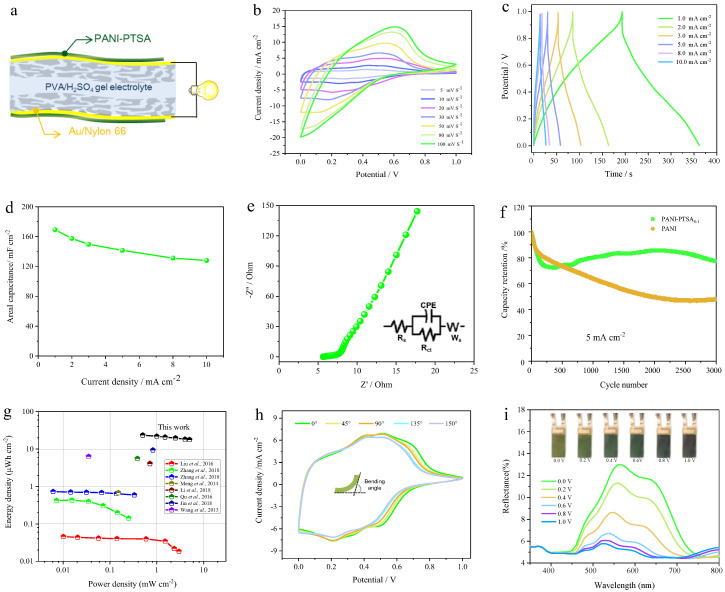
Performance and flexibility characterization of symmetric flexible ECSCs based on PANI-PTSA_8:1_ electrodes. (**a**) Schematic illustration of the device’s architecture. (**b**) CV curves measured at various scan rates. (**c**) GCD curves recorded at current densities ranging from 1 to 10 mA cm⁻^2^ reflect charge storage capabilities. (**d**) Rate performance of the ECSCs highlights capacitance retention at increasing current densities. (**e**) EIS plots reveal internal resistance and ion transport properties. (**f**) The cycling stability tested at a 50 mV s⁻^1^ scan rate confirms long-term durability. (**g**) The areal Ragone plot compares the energy and power densities of the devices to the reported literature [44,45,46,47,48,49,50]. (**h**) CV curves under different bending angles (0° to 150°) at 50 mV s⁻^1^. (i) Reflectance spectra at various applied voltages with inset images illustrate the device’s reversible electrochromic color changes from 0 to 1.0 V.

In order to further investigate the PANI-PTSA film electrode’s potential for practical applications, a flexible sandwich-shaped supercapacitor was built and assembled using two PANI-PTSA_8:1_ film electrodes coated in a PVA/H_2_SO_4_ electrolyte (Figure 5a). Here, we employ the PE film as a surface encapsulation film, which possesses considerable flexibility. The as-fabricated device does not need to include another separator layer, having a total thickness of around 390 μm (Figure S2). The CV curves under different scan rates, ranging from 5 to 100 mV s^−1^, and GCD curves under different areal current densities, ranging from 1 to 10 mA cm^−2^, of PANI-PTSA_8:1_-based supercapacitors are shown in Figure 5b,c. From the CV curves, the shape of the curves can be well maintained with an increase in the scan rate, illustrating good capacitive characteristic behavior [43]. The GCD curves demonstrate excellent charge–discharge behavior within the 0 to 1 V potential window for the devices. The areal specific capacitance calculated from the discharge curves reaches 169.2 mF cm⁻^2^ at a current density of 1 mA cm⁻^2^ and maintains 128.0 mF cm⁻^2^ even when the current density increases to 10 mA cm⁻^2^, corresponding to a high capacitance retention rate of 76%. Energy density (E, μWh cm⁻^2^) and power density (P, mW cm⁻^2^) are two key parameters used to evaluate the practical performance of energy storage devices. The Ragone plot in Figure 5g shows the areal energy and power densities of the supercapacitor. The device assembled with the PANI-PTSA electrode possesses a maximum areal energy of 23.5 µWh cm^−2^ at a power density of 0.5 mW cm^−2^ and a maximum areal power density of 1 mW cm^−2^ at an energy density of 21.9 µWh cm^−2^, whose values are better than those of previously published reports [44,45,46,47,48,49,50].

The EIS analysis for the PANI-PTSA devices is displayed in Figure 5e. Faster electrode kinetics and low internal resistance are associated with equivalent series resistances and charge transfer resistances of around 9.3 and 3 Ω, respectively. Long-term GCD tests at 5 mA cm⁻^2^ were conducted to evaluate the cycling stability of devices based on PANI and PANI-PTSA8:1 (Figure 5f). The PANI-PTSA8:1 electrode retains 79% of its initial capacitance after 3000 cycles, significantly outperforming the PANI electrode, which retains only 50%. To assess device flexibility, CV measurements were performed at various bending angles (0°, 45°, 90°, 135°, and 150°) at a scan rate of 50 mV s⁻^1^ (Figure 5h). The CV curves exhibit only slight decreases in current, demonstrating the device’s potential for application as a flexible multi-functional wearable device.

Furthermore, the reflectance spectrum of the device was measured at different applied voltages at 300–800 nm. The device shows visually distinguished color changes at different voltages, as shown in Figure 5i. The reflectance of the positive electrode of the device was gradually decreased when the applied voltage was switched from 0 to 1 V. Meanwhile, the gadget displayed a “light green→green→dark blue” color shift. The functioning statuses of the device can therefore be roughly observed in real time by corresponding color changes.

**Figure 6:**
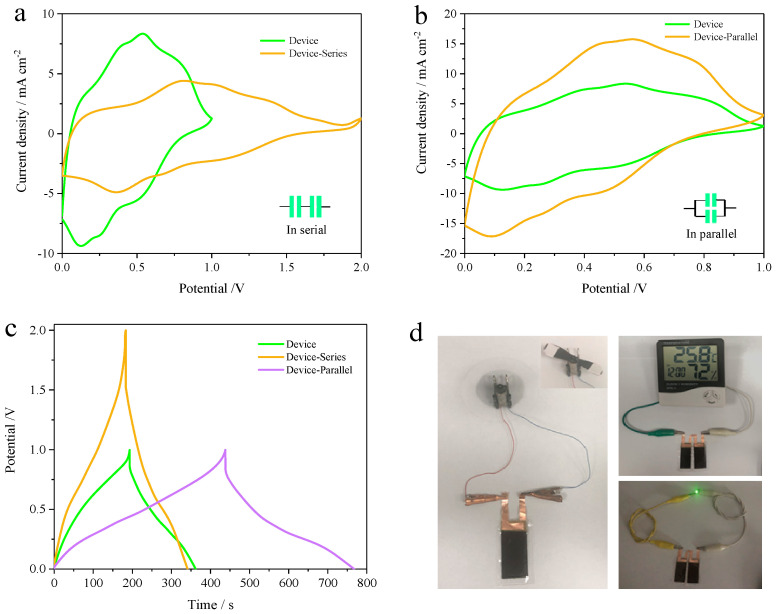
Electrochemical performance and practical demonstration of single and double asymmetrical supercapacitor (ASC) devices. (**a**,**b**) CV curves comparing single and double device configurations at various scan rates and (**c**) corresponding galvanostatic charge–discharge (GCD) curves illustrating their charge storage behavior. (**d**) Photographs of ASC devices powering a small electrical motor fan and illuminating green LEDs or an electronic clock.

To evaluate the integrability of the devices, they were easily connected in series or in parallel through continuous integration [51]. The CV curves show that connecting two devices in series effectively doubles the potential window from 1 V to 2 V, while a parallel connection increases the current output, as illustrated in Figure 6a,b. Correspondingly, the GCD profiles demonstrate an extended potential window and longer discharge times for devices connected in both series and parallel configurations, indicating excellent electrochemical performance and uniformity (Figure 6c). Finally, Figure 6d presents a digital image of the flexible devices, where two series-connected devices successfully power an LED timer, showcasing their promising potential for practical applications.

## 4. Conclusions

In summary, a special microsphere-like PANI nanocomposite was successfully prepared by mixing the aniline monomer and PTSA dopant, followed by the in situ electropolymerization of the PANI-PTSA film with a flexible Au/nylon 66 electrode, and was used to assemble a novel bi-functional device integrated with electrochromic and energy storage properties. The synergistic impact of the dopant and PANI was the primary cause of the device’s improved electrochemical performances. The distinctive aromatic ring structure of PTSA in the PANI-PTSA_8:1_ composite plays a significant synergistic role in enhancing specific capacitance. It also acts as an adhesive scaffold that helps regulate polymer morphology and improves cycling stability. Consequently, a paper-like symmetric supercapacitor was designed and fabricated using the optimized PANI-PTSA_8:1_ electrode and H_2_SO_4_/PVA polymer electrolyte. The resulting ECSCs exhibit excellent electrochemical performance, including a high specific capacitance of 169.2 mF cm⁻^2^ at 1 mA cm⁻^2^, a notable energy density of 23.5 μWh cm⁻^2^ at a power density of 0.5 mW cm⁻^2^, and excellent cycling stability with 79% capacitance retention after 3000 cycles. In addition, the smart ECSCs exhibit a reversible color change from yellow-green to deep blue during the charge–discharge process. The multi-functional device maintains high electrochemical performance even under bending and can power various electronic devices, such as small fans, LEDs, and electronic displays. Overall, these ECSCs, combining excellent color-switching and capacitive properties, demonstrate great potential for applications in smart wearable electronics based on PANI-based conductive polymers.

## Data Availability

The original contributions presented in this study are included in the article and Supplementary Materials. Further inquiries can be directed to the corresponding authors.

## Supplementary Materials

The following supporting information can be downloaded at: https://www.mdpi.com/article/10.3390/ma18122836/s1, Figure S1: (a) and (b) GCD curves of the areal capacitance and specific capacitance at various current densities for PANI film. Figure S2: The thickness of the ECSCs device. Figure S3: Picture of large Au/Nylon 66 film electrode.
