# Supplementary material for: Construction of Symmetric Flexible Electrochromic and Rechargeable Supercapacitors Based on a 1,3,6,8-Pyrenetetrasulfonic Acid Tetrasodium Salt-Loaded Polyaniline Nanostructured Film
Source: Materials (Basel). 2025 Jun 16;18(12):2836. doi: 10.3390/ma18122836 (PMC12195107; doi:10.3390/ma18122836)
Supplement: Supplementary file 1 [file materials-18-02836-s001.zip › materials-3681794-supplementary.pdf]

Supplementary Materials

Construction of Symmetric Flexible Electrochromic and Rechargeable Supercapacitors Based on a 1,3,6,8-Pyrenetetrasulfonic Acid Tetrasodium Salt-Loaded Polyaniline Nanostructured Film

Yi Wang [1,2], Ze Wang [1], Zilong Zhang [1], Yujie Yan [1], An Xie [1], Tong Feng [3,*] and Chunyang Jia [2,*]

[1]Key Laboratory of Functional Materials and Applications of Fujian Province, School of Materials Science and Engineering, Xiamen University of Technology, Xiamen 361024, China; yiwang@xmut.edu.cn (Y.W.); 2322161045@stu.xmut.edu.cn (Z.W.); 2222031544@stu.xmut.edu.cn (Z.Z.); yujieyan@xmut.edu.cn (Y.Y.); anxie@xmut.edu.cn (A.X.)

[2]National Key Laboratory of Electronic Thin Films and Integrated Devices, National Engi-neering Research Center of Electromagnetic Radlation Control Materials, School of Integrated Circuit Science and

Engineering, University of Electronic Science and Technology of China, Chengdu 610054, China

[3]School of Mechanical Electrical and Information Engineering, Xiamen Institute of Technology, Xiamen 361021, China

* Correspondence: fengtong@xit.edu.cn (T.F.); cyjia@uestc.edu.cn (C.J.)

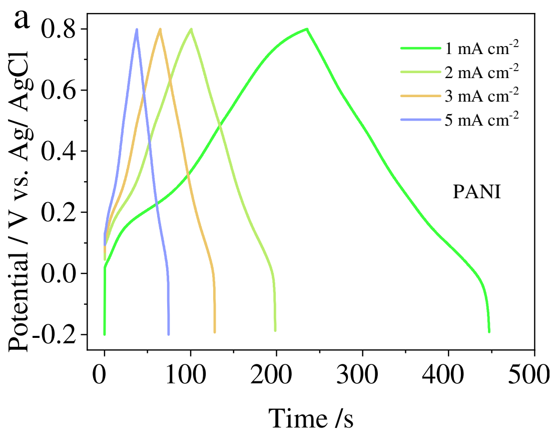

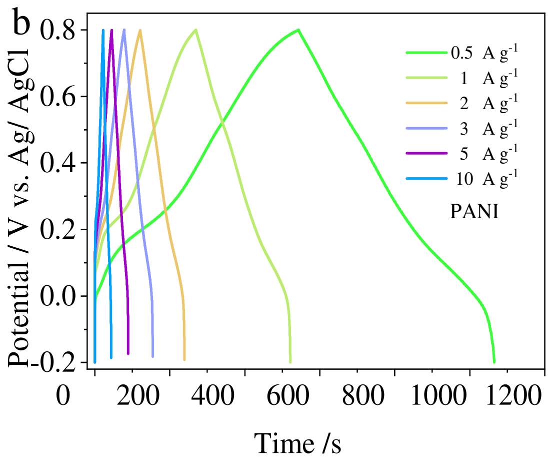

Figure S1. (a) and (b) GCD curves of the areal capacitance and specific capacitance at various current densities for PANI film.

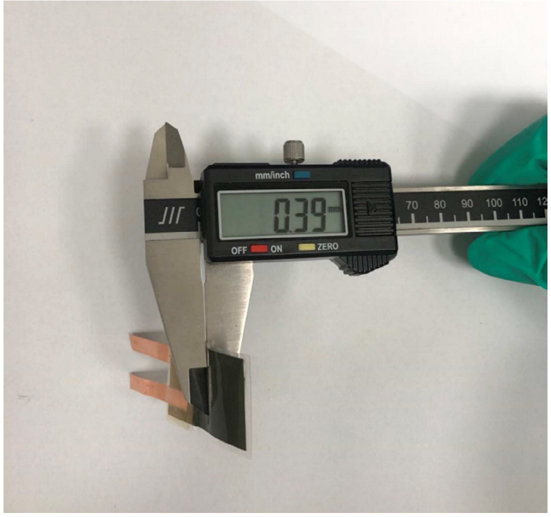

Figure S2. The thickness of the ECSCs device.

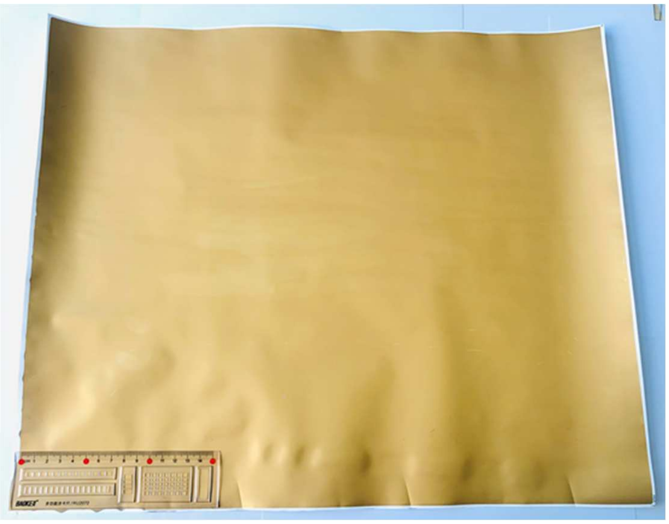

Figure S3. Picture of large Au/Nylon 66 film electrode.
